# The club cell circadian clock regulates temporal patterns in leukocyte trafficking in chronic allergic airways disease

**DOI:** 10.1016/j.mucimm.2026.100384

**Published:** 2026-10

**Authors:** Jafar Cain, Amlan Chakraborty, Vishvangi Deugi, Karolina Krakowiak, David A. Bechtold, Julie E. Gibbs, Hannah J. Durrington

**Affiliations:** a University of Manchester, School of Medical Sciences, Division of Diabetes, Endocrinology and Gastroenterology, United Kingdom; b Centre for Biological Timing, Faculty of Biology, Medicine and Health, University of Manchester, United Kingdom; c University of Manchester, School of Biological Sciences, Division of Immunology, Immunity to Infection and Respiratory Medicine, United Kingdom; d Manchester University NHS Foundation Trust, United Kingdom

**Keywords:** Asthma, Circadian, Trafficking, Inflammation, Epithelial cells

## Abstract

Patients with allergic asthma exhibit distinct temporal variations in symptoms and disease severity, which oscillate across the 24 h day. We have previously shown that the molecular circadian clock plays a critical role in temporally gating the response to allergen challenge in mice. Club cells, a subset of pulmonary bronchial epithelial cells, are implicated in the gating of time-dependent responses to type-1 inflammatory stimuli and trafficking of neutrophils to the lung. Using a club cell specific *Bmal1* knockout mouse (CCSP*-Bmal1* KO) we found that this is also true in the setting of chronic type-2 pulmonary inflammation, where eosinophil and T-helper 2 cell trafficking patterns were abolished in mice lacking a functional circadian clock within the lung. Notably, we find that circadian patterns in airway permeability are lost in CCSP*-Bmal1* KO mice, rendering the barrier equally permissible to trafficking cells at all times of day.

## Introduction

Asthma is a chronic inflammatory disease of the lungs affecting over 350 million individuals worldwide,^1^ commonly characterised by type-2 allergic airway inflammation, with T-helper (TH)2 cells and eosinophils being key drivers of disease. Many asthma symptoms exhibit temporal variations, typically being aggravated in the early hours of the morning2., 3.; implicating a role for the circadian clock in driving rhythmicity in asthma. Indeed, populations who experience circadian disruption, such as through night shiftwork, experience a heightened prevalence and severity of asthma.^4^ We previously found that the eosinophil chemoattractant eotaxin-1 peaks in concentration at 04:00 in sputum samples from patients with atopic asthma,^5^ coinciding with physiological nadirs in lung function.^5^, ^6^, 7. Moreover, it has been found that asthma exacerbations more commonly occur and are more likely to result in death at 04:00.2., 8 Although rhythmicity in asthma severity is a well-recognised and longstanding point of great interest in in the circadian biology of allergic airways disease,^9^, ^10^, ^11^ little is known about how the allergic inflammatory milieu of the lung changes over the course of the 24 h day. Gaining mechanistic insight into drivers of rhythmic inflammation offers the opportunity to develop new therapeutic approaches.

Most mammalian cells have a molecular circadian clock which produces rhythmic gene outputs in 24-h cycles. This molecular clock is centred on the actions of BMAL1 and CLOCK, transcription factors which drive the expression of clock-controlled genes.12., 13 REVERBs and RORs (including REVERBa, the protein coded for by the *Nr1d1* gene) are important clock outputs which form an accessory loop in the molecular circadian system and integrate the molecular clock with the immune system.^14^

Airway epithelial cells (AECs), when damaged by inflammatory stimuli (including house dust mite (HDM) allergens), release alarmins which trigger a multifaceted immune response involving subsets of T cells (TH2s, TH9s) and eosinophils. Eosinophils are key drivers of the pathophysiology in asthma. They enter the blood from the bone marrow and travel to the lung, releasing IL-13, which induces goblet cell metaplasia and mucus hypersecretion.^15^, ^16^ Moreover, IL-13 damages AECs, weakening the structural integrity of the epithelial barrier.^17^, 18., 19. It has been shown that epithelial barrier integrity at other mucosal sites such as the gut and cornea, relies on components of the core circadian clock (such as BMAL1) which control the temporal expression of tight junction genes and proteins such as ZO.1 and Occludin.20., 21., 22. The extent to which this applies also to the airway epithelium remains to be fully determined, and more so questions remain as to how such regulation of the airway epithelial barrier is coordinated across a heterogeneous tissue comprising many different cell types, and how this changes under inflammatory conditions.

Club cells, non-ciliated secretory epithelial cells in the small airways, have been identified as key cell types that under homeostatic conditions maintain the health of the airway barrier.23., 24 In allergic inflammatory conditions however, club cells contribute to worsening pathologies in patients with asthma.23., 24 Club cellshave also previously been identified as a population within the airway epithelium acting as local pacemakers,^25^, ^26^ coordinating and synchronising the actions of a wide range of local cell types.27., 28., 29, 30., 31. Disrupting the circadian clock in CCSP-expressing cells (predominantly club cells) via (CCSP^icre^) *Bmal1* ablation leads to a loss of time-of-day variations in type-1 inflammatory lung disease models, and exacerbates disease severity.^25^, ^26^ However, the role of the club cell clock in coordinating rhythmicity in type-2 inflammatory diseases such as asthma remains to be explored. Moreover, the potential role of club cells to act as pacemakers for other aspects of mucosal barrier biology, including rhythms in barrier permeability, has not yet been addressed.

Utilising a mouse model of chronic allergic airways disease (AAD, reminiscent of asthma), we show that the club cell circadian clock is responsible for coordinating the time-of-day dependent accumulation of leukocytes in the lung and airways. Furthermore, we demonstrate that a functional molecular clock within the club cell is vital for maintaining physiological time-of-day dependent variations in airway barrier integrity. In addition, we demonstrate for the first time that AAD-associated loss of barrier integrity can be restored through modulating the circadian clock in the airway epithelium.

## Methods

### Animals

Mice were maintained in the University of Manchester Biological Services Facility. All procedures were approved by the University of Manchester Animal Welfare and Ethical Review Body and carried out according to the Animals (Scientific Procedures) Act 1986. Mice were group housed under a 12 h: 12 h light/dark cycle with ad libitum access to food and water. Lights on (start of the rest phase) is referred to as Zeitgeber Time (ZT)0 and lights off (start of the active phase) as ZT12. C57BL6 mice were purchased from ENVIGO (*Huntingdon, United Kingdom*). CCSP-*Bmal1* KO mice were bred in house as described elsewhere.^25^ These were bred as homozygous for *Bmal1*^flox^ (*Bmal1*^flox/flox^) with either one copy of the iCre (CCSP^iCre^; CCSP-*Bmal1* KO) or no copies (wild type littermates).

To induce chronic AAD female mice (aged 6 to 8 weeks) were dosed with HDM (Citeq; batches 15 J02/20A05)^32^ or vehicle (phosphate buffered saline (PBS)) three times per week (Monday, Wednesday, Friday) for a period of 5 weeks. Female mice were used here exclusively due to their greater propensity for experiencing robust allergic inflammatory responses compared to male mice.^33^ For in-vitro ALI experiments, tissue from male mice of the CCSP-*Bmal1* KO strain was used. We note that the molecular circadian clock is mechanistically preserved between sexes.^34^, ^35^ Intranasal HDM dosing was performed at ZT11 (6 pm) as our previous work elucidated that dosing at this time produces robust inflammatory responses.^32^ Sample collection began on Day 36 (Monday of week 6), in order to leave enough time from the final dose of HDM to separate dose-response effects from the effects of circadian regulation which are expected to persist even in the absence of immediate intranasal HDM stimulation.

At the end of the protocol, blood, broncho-alveolar fluid (BALF), lung and spleen tissue were collected. For in-vivo SR9009 dosing mice were intraperitoneally administered 100 mg/kg SR9009 (dissolved in DMSO) or an equivalent volume of DMSO every day at ZT6 for the final 14 days of the AAD protocol.

### Tissue preparations for flow cytometry

Lung, BALF, and spleen samples were processed as previously described.^25^, ^26^, 36. Blood samples were collected via tail-bleeds in EDTA coated tubes and processed according to an established protocol.^37^

### Flow cytometry staining

Cells were stained with a live/dead marker (LIVE/DEAD Blue, Invitrogen, 1:1000) in PBS and Fc receptors were blocked (1:100 anti-CD16/32) before application of cell surface antibody cocktails (described below). After washing, cells were fixed in 50 μl 4% PFA for 30 min before being permeabilised, if required, in 100 μl permeabilisation buffer (True Nuclear Fix Perm Kit, Biolegend). Following permeabilisation, cells were stained in the relevant intracellular staining cocktail (1200 in 100 μl described below) for 1 h. After final washing, cell pellets were resuspended in 150 μl FACS buffer (PBS with 2% FBS and 2 mM EDTA) and analysis was carried out on a BD Fortessa flow cytometer. ArC amine reactive compensation beads and OneComp ebeads (ThermoFisher) were utilised to facilitate compensation of live/dead staining and single colour staining respectively. Cell populations were subsequently analysed using the FlowJo software (v10.10.0).

Cells were gated as follows: Eosinophils (LY6G-, Siglec-F+, CD11c-, CD11b+), Neutrophils (LY6G+, CD11b+), TH2 (CD4+, TCRβ+, CD8-, GATA3+, FOXP3-, PU.1-), TH9 (CD4+, TCRβ+, CD8-, GATA3-, FOXP3-, PU.1+), T-reg (CD4+, TCRβ+, CD8-, CD25+, FOXP3+), B-cells (CD45+, B220+).

### Flow cytometry antibodies

CD4-AF700 (*Biolegend,* GK1.5), CD8-BV785 (*Biolegend, 53–6.7),* TCRβ-eF506 (*Invitrogen, H57–597),* CD25-AF488 (*Biolegend, PC61.5),* FOXP3-eF450 (*Invitrogen, FJK-16* *s),* PU.1-PE (*Biolegend, 7C2C34),* GATA3-eF660 (*Invitrogen, TWAJ),* CD11c-PerCp.Cy5.5 (*Invitrogen, M1/70),* CD11b-BV510 *(BD Horizon, HL3),* Siglec-F-PE (*BD Pharmingen, E50–2440),* LY6G-eF450 (*Invitrogen*, *1A8*-*LY6G*), B220-APC eF780 (*Invitrogen, RA3.6B2)*.

### Bioplex assay

BALF was analysed using Bio-Plex Pro ™ Mouse Chemokine Panel 23-Plex (Bio-Rad, #M60009RDPD) as per the manufacturer's instructions.

### RT-qPCR

RNA was isolated from lung tissue samples, and RT-qPCR performed as previously described.^32^ Briefly, cDNA was synthesised using GoScript™ Reverse Transcription Mix (Promega), before RT-qPCR reactions were performed using KAPA SYBR FAST qPCR Master Mix (ROCHE) on an Applied Biosystems StepOnePlus Real-Time PCR system. Minimum 260/280 values for RNA sample input was assessed prior to cDNA synthesis and samples above 1.80 were deemed suitable for subsequent qPCR analysis.

Data from qPCR assays were normalised against the housekeeping gene *Hprt*. Primer sequences used were as follows: *Zo.1 F (*GCCGCTAAGAGCACAGCA*), Zo.1 R* (TCCCCACTCTGAAAATGAGGA), *Ocln* F (TTGAAAGTCCACCTCCTTACAGA), *Ocln R* (CCGGATAAAAAGAGTACGCTGG), *Hprt* F (GTTGGATACAGGCCAGACTTTGTTG) *Hprt* R (GATTCAACTTGCGCTCATCTTAGGC).

### Immunoblotting

Mouse lungs were weighed and approximately 30–35 mg of tissue was lysed using 0.5 ml T-Per™ tissue protein extraction reagent (Thermo Scientific) supplemented with protease and phosphatase inhibitor cocktail (cOmplete™, Mini, EDTA-free Protease Inhibitor Cocktail, MilliporeSigma) using a lysing matrix D on a homogenizer. Proteins were isolated from the lysate supernatant and quantified using Pierce BCA assay, and 50 μg of protein was heated at 95 °C for 5 min in Laemmli SDS sample reducing buffer (Thermo Scientific) and loaded into Any kD™ Mini-PROTEAN® TGX™ Precast Protein Gels (BioRad). Gels were run using premixed electrophoresis buffer (BioRad) at 100 V for an hour. Proteins were transferred onto Trans- Blot Turbo Mini 0.2 μm PVDF transfer packs (BioRad) on a Trans-Blot Turbo transfer system for 10 min. The membrane was blocked at room temperature for 2 h with 5% skim-milk in Tris-Buffered Saline with 0.1% Tween-20 (blocking buffer) under gentle shaking. The membranes were then incubated overnight at 4 °C with primary antibodies in blocking buffer followed by washing in TBS-T buffer and incubating for 2 h at room temperature with the secondary antibody. Primary antibodies used were: ZO-1 (21773–1-AP, *Proteintech*, 1:5000), Occludin (27260–1-AP, *Proteintech*, 1:10000) and Alpha Tubulin (T6199, Sigma-Aldrich, 1:5000), REVERBα (14506–1-AP, *Proteintech*, 1:1000) and secondary antibody anti-Rabbit IgG HRP conjugated (7074, *Cell Signalling Technology*, 12000). HRP conjugated secondary antibody was detected using the Clarity Western ECL substrate detection kit (BioRad, 1705061) and quantified by densitometry with a ChemiDoc MP Imaging System and Image Lab v.6.0 software (both from Bio-Rad Laboratories, Hercules, CA, USA). The densitometry values were then expressed relative to the value from the HDM-challenged group. Raw unprocessed images of all immunoblots can be found in Supplementary Fig. S7.

### Histology

The upper and lower right lobes of each lung were fixed overnight in 10% neutral buffered formalin before processing and paraffin embedding. 5 μm sections were cut on a microtome and mounted onto slides. Sections were stained with Haematoxylin & Eosin, Alcian Blue – Periodic Acid Schiff, or Masson's Trichrome using established protocols.

Immunohistochemical staining was performed after heat induced epitope retrieval in citrate buffer (pH 6.0, 60 °C, 30mins) using primary antibodies for ZO.1(1:3000, ABCAM), OCLN (1:3000, ABCAM), TSLP (1:4000, EMD Millipore Corporation), and secondary Anti-Rabbit HRP labelled antibody (DAKO EnVision+ System).^38^ Detection of positive staining regions was then performed using DAB+ Chromogen (DAKO REAL EnVision). Slides were then counterstained with Harris' Haematoxylin and blued with Scott's Tap Water (pH 7.5).

### ELISA

Sandwich ELISAs were performed to detect and quantify serum IgE (EMIGHE, *Thermofisher)* and BALF Albumin (ab108792, *ABCAM)* both according to the manufacturer instructions.

### Air-liquid interface cell culture

Tracheal epithelial cells from male CCSP-*Bmal1* KO and wild-type littermate mice (10–13 weeks) were cultured as previously described, using Pneumacult-ALI medium (*STEMCELL Technologies*).^39^ Cells were synchronised with dexamethasone (200 ng/ml, 2 h) before transepithelial electrical resistance (TEER) measurements every 4 h for 96 h using an electrovoltmeter, in the presence/absence of IL-13 (20 ng/ml) and/or SR9009 (5 μM) or DMSO control.

### Statistical analysis

Statistical analysis was performed using GraphPad Prism software version 10. Two-way, and Three-way ANOVAs were used to determine significance of variables, with post-hoc Sidak's/ Dunnett's multiple comparison tests as appropriate. A *p*-value ≤0.05 was considered statistically significant. Cosinor analysis was conducted in DiscoRhythm v1.2.1.^40^ Throughout, the outcomes of statistical testing were shown as: *P < 0.05, **P < 0.01, ***P < 0.001, ****P < 0.0001.

## Results

### Leukocyte populations vary by time-of-day in chronic allergic airways disease (AAD)

Using a chronic model of AAD, we sought to establish time-of-day dependent patterns in leukocyte accumulation in the blood, BALF, lungs, and spleen. Tissue was collected at ZT0 (time of lights on and start of the rest phase), ZT6 (mid-rest phase), ZT12 (lights off and start of the active phase) and ZT18 (mid-active phase). After 5 weeks of sensitisation with HDM (batch 15 J02) (Fig. 1A), circulating blood eosinophils were quantified by flow cytometry (Siglec-F+, CD11b+) in HDM-exposed mice and PBS-treated controls over the 40-h following the final exposure (Fig. 1B). Under normal conditions, circulating eosinophils were elevated at ZT6 compared to all other time points, a pattern significantly amplified in the presence of AAD (Fig. 1B). We observed the same time-of-day oscillations in both PBS and HDM-treated mice, suggesting that this rhythmicity in circulating eosinophils is present under homeostatic conditions, and then amplified in the presence of AAD (Fig. 1B).

**Fig. 1:**
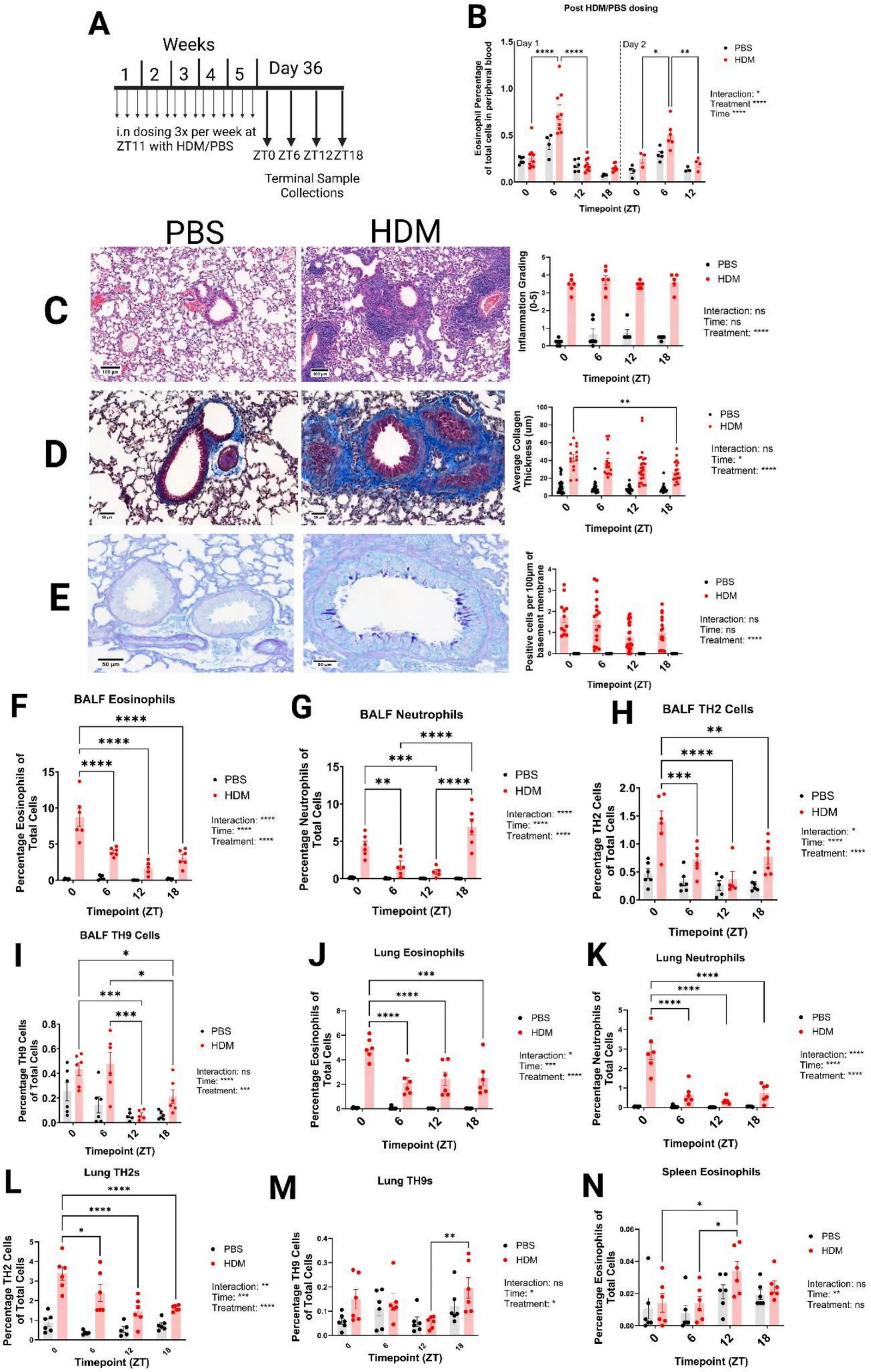
Leukocyte populations vary by time-of-day in chronic allergic airways disease (AAD) (A) – Schematic overview of 5-week dosing protocol and sampling timepoints. (B) – Quantification of eosinophils in peripheral blood samples across 7 consecutive 6-hourly timepoints following the final dose of HDM/PBS. (C) - Representative images of haematoxylin and eosin-stained lung sections from PBS/HDM challenged mice on day 36 of the chronic AAD protocol and quantification of inflammation scoring across all 6-hourly timepoints. (D) - Representative images of Masson's Trichrome-stained lung sections from PBS/HDM challenged mice on day 36 of the chronic AAD protocol and quantification of peribronchiolar collagen deposition across all 6-hourly timepoints. (E) - Representative images of alcian blue periodic acid schiff stained lung sections from PBS/HDM challenged mice on day 36 of the chronic AAD protocol and quantification of positive stained cells per 100 μm of basement membrane across all 6-hourly timepoints. (F – N) – Quantification of immune cell subsets in BALF, and Lung sampled across 4 6-hourly timepoints on day 36 of the chronic HDM exposure protocol. (B—N) – all data presented as mean +/− SEM analysed by two-way ANOVA with post-hoc Šidak's multiple comparison tests. *N* = 3–9. First level output of two-way ANOVA shown as Interaction / Time / Treatment. (For interpretation of the references to colour in this figure legend, the reader is referred to the web version of this article.)

In the lung, chronic HDM exposure induced gross pathological changes which were stable across timepoints (Fig. 1C–E). Both haematoxylin and eosin (Fig. 1C), and Alcian Blue Periodic Acid Schiff (Fig. 1FE staining revealed treatment-dependent inflammatory changes in the lung tissue however this did not vary significantly by time-of-day. Due to the chronic nature of the protocol, we did not anticipate finding significant time-dependent differences in peribronchiolar collagen deposition, although analysis suggested that in ZT0 samples there was more deposition compared to ZT18 samples (Fig. 1D).

Under homeostatic conditions, BALF typically consists of 85–95% alveolar macrophages, with granulocyte populations, including eosinophils, constituting only a minor fraction of total cell counts (<1%). In HDM models of allergic airways disease eosinophil populations expand significantly and contemporaneous increases in other leukocyte populations (TH cells and neutrophils) are also expected.^41^, ^42^ In our chronic HDM model, exposure to allergen precipitated both an absolute and a relative increase in eosinophil, neutrophil, TH2, and TH9 cell populations in the airways (measured in BALF), especially during the rest phase (ZT0, ZT6) (Fig. 1F–I; Sup Fig. S1A - F). Likewise, in the lung, there was a strong treatment effect associated with HDM exposure, with greater populations of eosinophils and TH2 cells occurring during the rest period (Fig. 1J–M), however this was not found to apply to T-reg cells (Sup Fig. S1G). Additionally, splenic eosinophil populations in both PBS and HDM-treated mice were elevated at ZT12 (Fig. 1N). In lung and spleen tissue, cell populations were analysed as a percentage of total cells, rather than in absolute terms, to account for normal biological differences in tissue size and mass.

Overall, our findings show that despite all HDM-exposed mice reaching comparable levels of gross pathology, distinct compartment specific time-of-day patterns in leukocyte populations exist. In the blood, eosinophils exhibit daily oscillatory cycles, being most abundant at ZT6. Inflammatory cell populations in the lung and BALF peak at ZT0, and trough at ZT12.

### Airway cytokines vary by time-of-day in allergic airways disease

Having observed daily variation in accumulation of pro-inflammatory leukocytes within the allergically inflamed lung we next examined whether cytokine signals associated with these cells exhibited time-of-day variation. We find that in HDM (batch 15 J02)-exposed mice significant time-of-day dependent variations exist in a range of BALF cytokines (Fig. 2A).

**Fig. 2:**
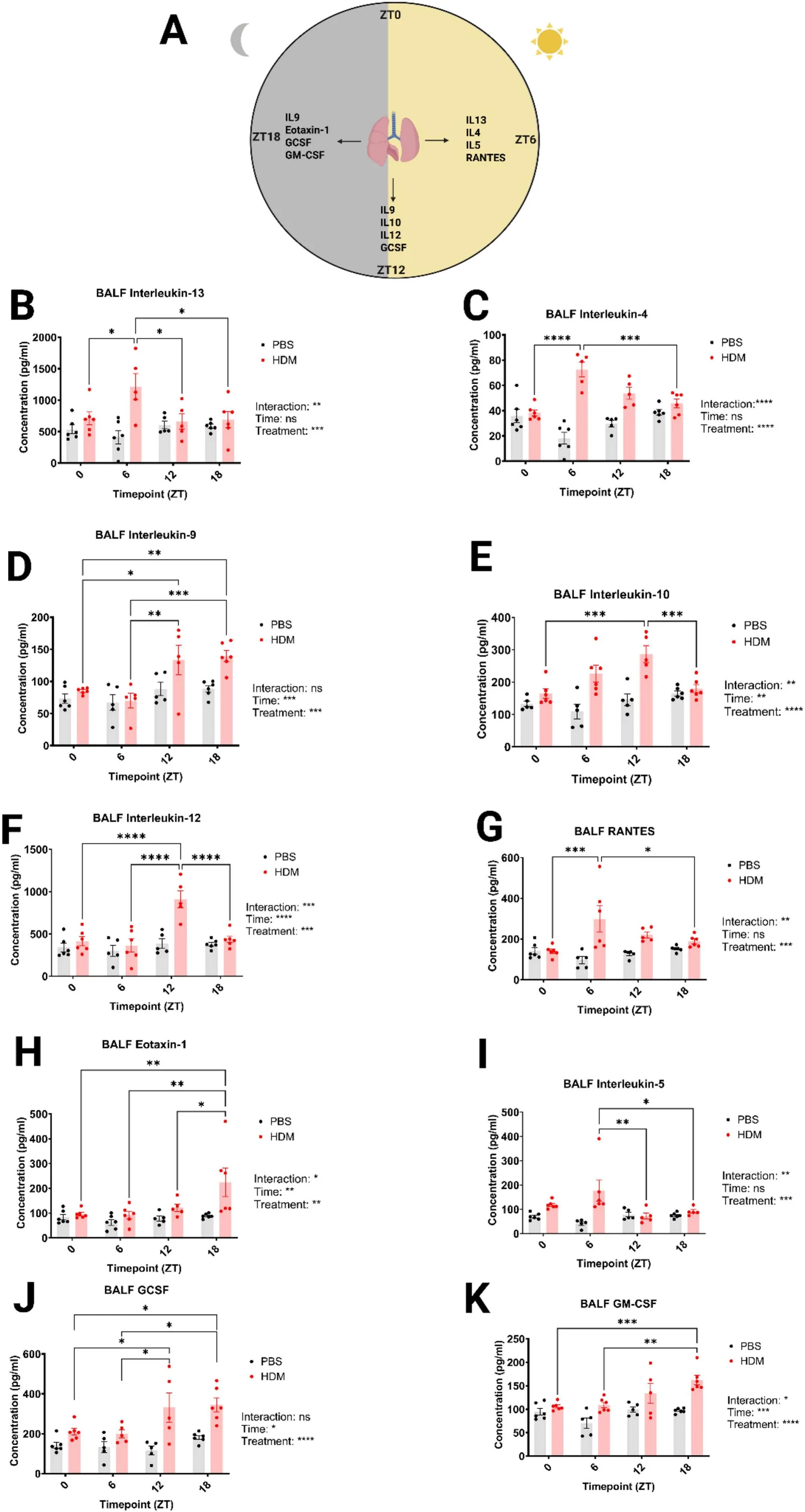
BALF cytokine concentrations vary by time-of-day in the HDM model of allergic airways disease (A) – Schematic outline of the time-of-day at which important BALF cytokines are significantly elevated. At ZT6, cytokines such as IL-13, IL-4, IL-5, and RANTES, associated with TH-cell activity peak in the BALF of HDM-exposed mice. At ZT12 IL-9, IL-10, and IL-12 are found to peak in their respective concentrations in the BALF of HDM-exposed mice. Eotaxin-1, GCSF, and GM-CSF are found to reach their peak concentrations in the BALF at ZT18, a timepoint in which IL-9 is also elevated. (B—K) - Quantification of cytokines present in BALF samples of HDM/PBS treated mice taken at 4 6-hourly intervals on day 36 of the chronic HDM exposure protocol. (B—K) - all data presented as mean +/− SEM analysed by two-way ANOVA with post-hoc Šidak's multiple comparison tests. *N* = 5–6. First level output of two-way ANOVA shown as Interaction / Time / Treatment.

Whilst TH2 cells peak in the BALF and lung of HDM-exposed mice at ZT0 (Fig. 1I, M), TH2 cytokines, namely IL-4, IL-13 and IL-5, were most elevated at ZT6 (Fig. 2B–D). Eotaxin-1, a key eosinophil chemoattractant, peaked at ZT18 (Fig. 2J), 6-h preceding the peak in BALF eosinophil populations (Fig. 1G). IL-9 levels were significantly raised during the mouse active phase (ZT12/18) (Fig. 2D), a finding that correlates with patterns observed in TH9 cell numbers in the BALF (Fig. 1J).

Whilst some elevation in type-1 cytokines was expected in this model of AAD (Supp Fig. S1 K - M), only IL-12, predominantly produced by antigen presenting cells, exhibited temporal variation, being most elevated at ZT12 in the BALF of HDM-exposed mice (Fig. 2F).

Leukopoietic cytokines were found to be elevated in BALF samples from HDM-exposed mice compared to PBS controls and significant time-of-day dependent variations were also observed in the concentration of GM-CSF, IL-5, and GCSF (Fig. 2I–K). GCSF and GM-CSF were found to peak in concentration during the mouse active phase (Fig. 2K, J), whilst IL-5 peaked at ZT6 in the BALF of HDM-exposed mice (Fig. 2I) Overall, these findings reveal that in the allergically inflamed lung, distinct time-of-day dependent variations in cytokine production exist varying according to cytokine type.

### In AAD the club cell circadian clock regulates time-of-day changes in immune cell populations within the lung and airway

Building on our observations that HDM-exposure was associated with time-of-day dependent patterns of immune cell accumulation in the BALF, we repeated the chronic HDM model (here with HDM batch 20A05) in mice lacking a functional circadian clock in the club cells of the airway epithelium, CCSP-*Bmal1* KO mice.

Histological analyses of lung tissue assessed mucin production, immune cell infiltration, and peribronchiolar collagen deposition (Fig. 3A–C). While no time-of-day variations were found in mucin staining in HDM-exposed WT littermate control mice (*Bmal1*^flox/flox^), CCSP-*Bmal1* KO mice showed increased mucin at ZT12 compared to ZT0 (Fig. 3A).

**Fig. 3:**
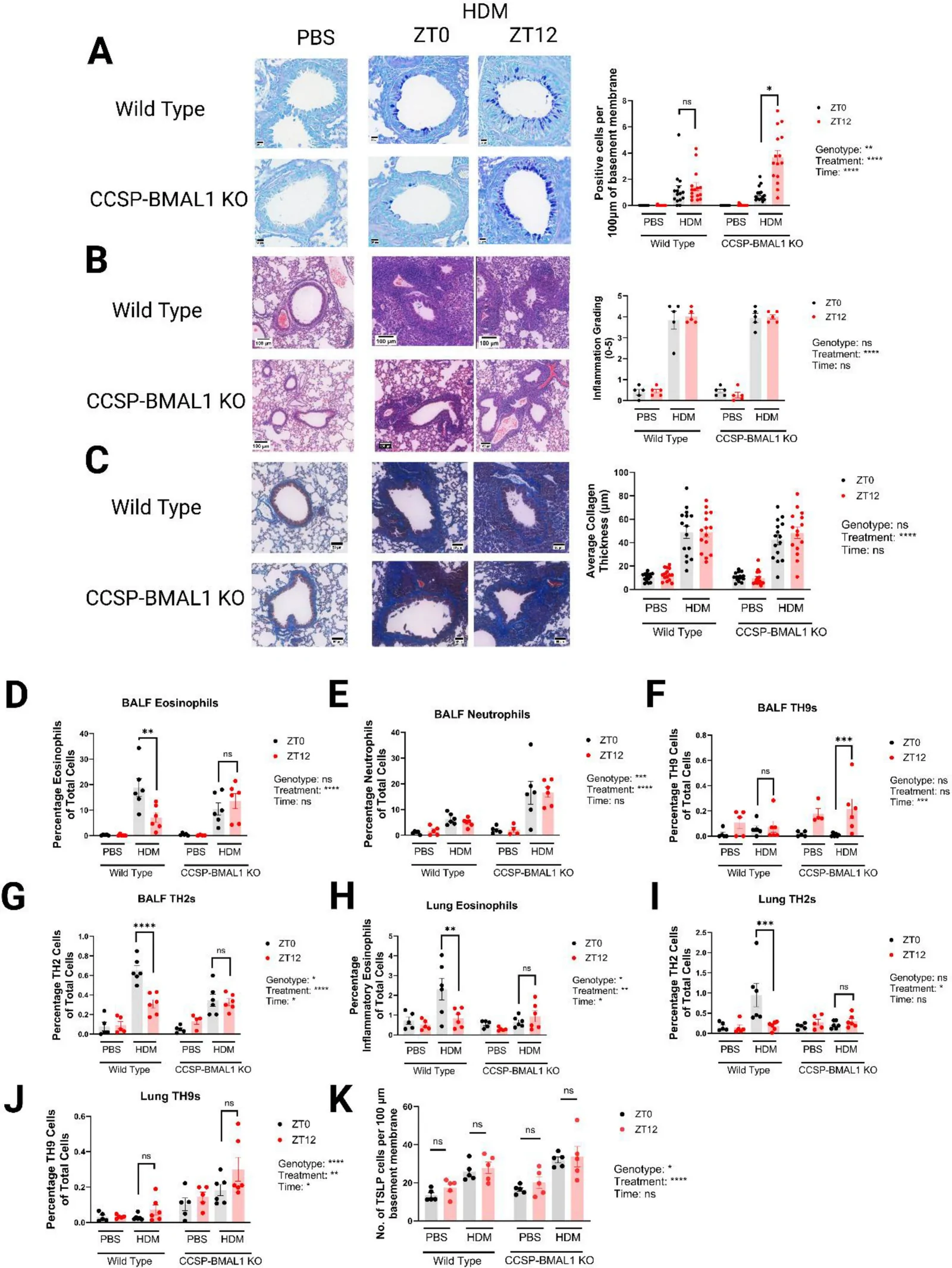
The club cell circadian clock is responsible for gating time-of-day differences in immune cell accumulation in the lung and BALF of HDM-exposed mice (A) – Representative images and quantification of positive mucin staining in AB-PAS stained FFPE lung sections taken at ZT0 and ZT12 from HDM/PBS treated CCSP-BMAL1 KO mice and wild type littermate controls, shown as number of positive cells per 100 μm of basement membrane. (B) – Representative images and quantification of H&E stained FFPE lung sections, taken at ZT0 and ZT12 from HDM/PBS treated CCSP-BMAL1 KO mice and wild type littermate controls, according to the scale detailed in Denny et al. 2015. (C) – Representative images and quantification of the extent of peribronchiolar collagen deposition in FFPE lung sections, taken at ZT0 and ZT12 from HDM/PBS treated CCSP-BMAL1 KO mice and wild type littermate controls, measured in Aperio Case Viewer. (E – K) – Quantification of immune cell subsets in BALF and Lung of HDM/PBS treated CCSP-BMAL1 KO mice and wildtype littermate controls, sampled at ZT0 and ZT12 on day 36 of the chronic HDM exposure protocol. (L) Quantification of TSLP positive cells per 100 μm of basement membrane. (A-L) – all data presented as mean +/− SEM analysed by three-way ANOVA with post-hoc Šidak's multiple comparison tests. N = 5–16. First level output of three-way ANOVA shown as Genotype / Treatment / Time.

We noted that IgE concentrations in the serum varied by time-of-day, being significantly elevated at ZT12 compared to ZT0 in WT controls, but not in HDM-exposed CCSP-*Bmal1* KO mice where IgE was significantly reduced (Supp Fig. S2J). B-cell numbers in peripheral blood, did not vary by time-of-day in WT and CCSP-*Bmal1* KO mice (Supp Fig. S2K) suggesting that the variance in IgE concentration is linked to functional activity rather than increases in the size of the B-cell population.

There was an increase in BALF total cell numbers after HDM-exposure in both WT and CCSP-*Bmal1* KO mice, which did not vary between timepoints (Supp Fig. S2A). The composition of BALF samples however differed significantly between genotypes and timepoints. In WT mice, both BALF eosinophils and TH2 cell populations were raised at ZT0 compared to ZT12 (as found previously, Fig. 1G, I), a phenomenon that was lost in CCSP-*Bmal1* KO mice (Fig. 3D, G). Furthermore, we found that the CCSP-*Bmal1* KO genotype was associated with increased BALF neutrophilia, in response to HDM-exposure, compared to WT mice, regardless of time (Fig. 3E). We noted also that the CCSP-*Bmal1* KO genotype was associated with increased TH9 cell populations in both the lung and BALF (Fig. 3F, J). TH9 cell populations were most elevated at ZT12 in HDM-exposed CCSP-*Bmal1* KO mice (Fig. 3F, J), something which was not seen in WT HDM-exposed mice in this experiment (Fig. 3F, J), nor in previous experiments with WT mice, where ZT0 and ZT6 had been associated with increases in TH9 cell populations (Fig. 1J).

Flow cytometric analysis of lung samples revealed that eosinophils, along with TH2 cells, were significantly raised in HDM-exposed WT mice at ZT0, compared to ZT12 (Fig. 3H, I). This temporal variation was lost in samples from CCSP-*Bmal1* KO mice (Fig. 3H, I). Further examination of eosinophil populations revealed that this time-of-day difference was driven by changes in CD11c^mid^ (inflammatory^43^) eosinophils (Fig. 3I) rather than a baseline expansion of CD11c^low^ (tissue resident^43^) eosinophils (Sup Fig. S2 F). Curiously, within the lung we again observed that the CCSP-*Bmal1* KO genotype, regardless of time, was associated with increases in TH9 cell populations, compared to WT littermate controls (Fig. 3J). To assess whether the time-of-day dependent variations observed here were due to temporal variation in epithelial alarmin production, we stained lung sections from PBS and HDM treated WT and CCSP-BMAL1 KO mice to detect the presence of TSLP (Fig. 3K). We found that whilst HDM-exposure was associated with greater levels of airway TSLP detection, this did not vary between ZT0 and ZT12, nor did it vary significantly between genotypes (Fig. 3K).

### A functioning club cell circadian clock is needed to maintain time-of-day dependent variations in several BALF cytokines

Quantification of cytokines within the BALF following HDM (batch 20A05) exposure revealed that IL-5, a key marker of type-2 inflammation, was elevated in HDM-exposed WT mice at ZT0 compared to ZT12, a finding not observed in HDM-exposed CCSP-*Bmal1* KO mice (Fig. 4A). Furthermore, an overall, genotype-associated increase in IL-9 was observed in CCSP-*Bmal1* KO mice, regardless of time (Fig. 4B). This aligns with our observation that TH9 cell populations were increased in the lungs and BALF of these same mice (Fig. 3F, J).

**Fig. 4:**
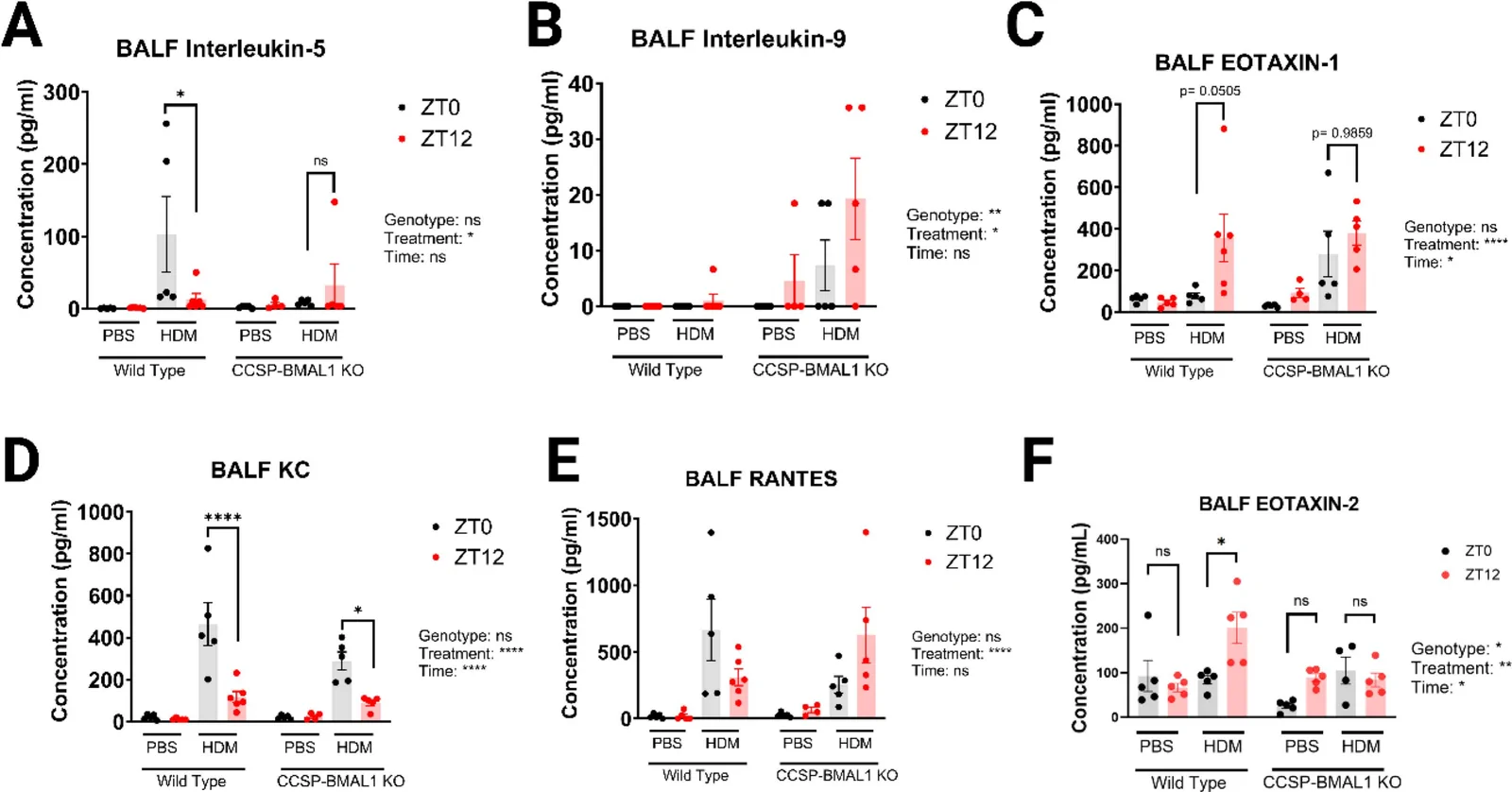
A functioning club cell circadian clock is needed to maintain time-of-day dependent variations in a number of BALF cytokines (A-F) – Quantification of cytokines present in BALF samples of HDM/PBS treated CCSP-BMAL1 KO mice and wild type littermate controls taken at ZT0 and ZT12 on day 36 of the chronic HDM exposure protocol. All data presented as mean +/− SEM analysed by three-way ANOVA with post-hoc Šidak's multiple comparison tests. N = 5–6. First level output of three-way ANOVA shown as Genotype / Time / Treatment.

Crucially, eotaxin-1 and eotaxin-2 concentrations did exhibit time-of-day variation according to the three-way ANOVA, however post hoc comparisons demonstrated that this temporal variation, for eotaxin-1, was just outside the threshold for significance (*p* = 0.0505) in WT HDM-exposed mice (Fig. 4C, F). HDM-exposed CCSP-*Bmal1* KO mice however did not show any time-of-day variation in eotaxin-1 or eotaxin-2 (Fig. 4C, F).

### The club cell circadian clock is essential for the temporal regulation of airway barrier integrity

Based on our observations that leukocyte populations in the BALF exhibit time-of-day variations, we sought to assess whether this could be attributed to variability in epithelial tight junction function and if this could further be related to the club cell circadian clock. Immunohistochemical investigation of WT mice revealed that tight junction proteins ZO.1 and Occludin were most abundant in PBS-treated mice at ZT6, and expression was reduced following HDM exposure, implicating loss of barrier integrity (Supp Fig. S4A–J). Through RT-qPCR analysis of lung tissue from naïve mice at ZT6 and ZT18, we found that gene expression of *Zo.1* and *Ocln* varies by time-of-day in WT mice (Fig. 5A, B) but this variation was strikingly lost when *Bmal1* was ablated from club cells (Fig. 5A, B). Building on this finding we isolated primary tracheal epithelial cells and differentiated them into a monolayer of mature epithelial cells using an air liquid interface (ALI) protocol (Fig. 5C).We then tested the rhythmicity of the transepithelial electrical resistance (TEER; a measure of tight junction integrity) across ALI cell membranes grown from WT and CCSP-*Bmal1* KO derived cells (Fig. 5D). Following synchronisation with dexamethasone (dex), cosinor analysis revealed significant rhythmicity in the TEER of WT ALI cell cultures (Fig. 5D). In contrast, ALI cultures from CCSP-*Bmal1* KO mice did not exhibit temporal variation in TEER measurements (Fig. 5D).

**Fig. 5:**
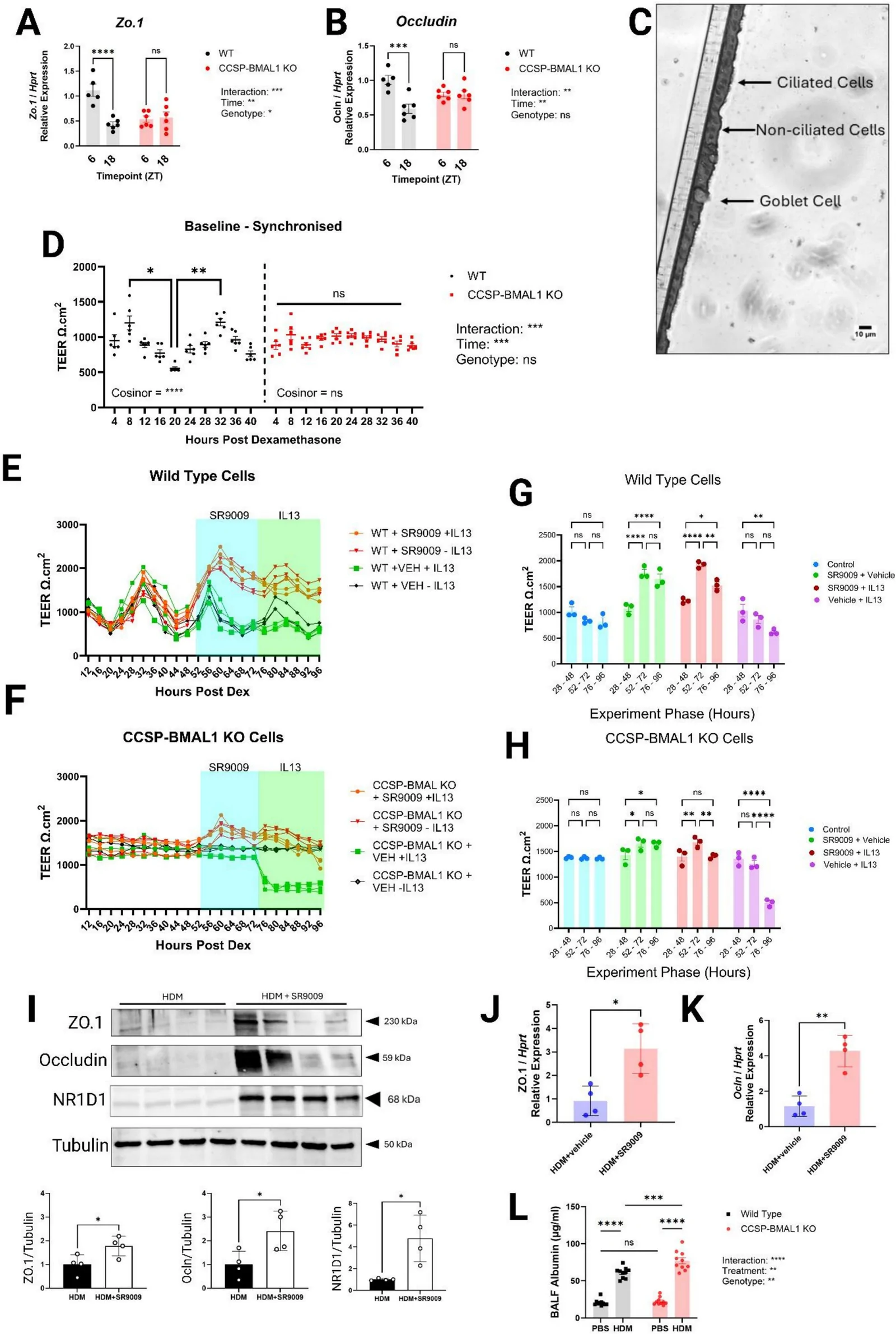
The club cell circadian clock is essential for the temporal regulation of airway barrier integrity (A) – qPCR data showing the relative expression of tight junction gene *Zo.1* at ZT6 and ZT18 in whole lung samples taken from male CCSP-BMAL1 KO mice and wild type littermate controls. (B) - qPCR data showing the relative expression of tight junction gene *Ocln* at ZT6 and ZT18 in whole lung samples taken from male CCSP-BMAL1 KO mice and wild type littermate controls. (A-B) –qPCR data was normalised to the wild type ZT6 group, and relative to the expression of housekeeping gene *Hrpt,* shown as mean +/− SEM, analysed by two-way ANOVA with post-hoc Sidak's multiple comparison tests, *n* = 5–6. First level output of two-way ANOVA shown as Interaction / Time / Genotype. (C) – Representative image of an FFPE section of Air-Liquid interface cultured epithelial cells taken using a EVOS M5000 light microscope. (D) – Trans-epithelial Electrical Resistance measurements taken from wild type and CCSP-BMAL1 air-liquid interface airway epithelial cell cultures every 4 h for 40 h post-synchronisation of internal clocks using dexamethasone. Data shown as mean +/− SEM, *n* = 6 per timepoint per genotype, analysed by two-way repeated measurements ANOVA with post-hoc Tukey's *t*-tests. First level output of two-way ANOVA shown as Interaction / Genotype / Time. Rhythmicity was assessed using Cosinor analysis in DiscoRhythm. (*E*-F) - Trans-epithelial Electrical Resistance measurements taken from wild type (E) and CCSP-BMAL1 KO (F) air-liquid interface airway epithelial cell cultures every 4 h from 12 h post-synchronisation of internal clocks using dexamethasone to 96 h post-synchronisation. At hour 48 cultures were dosed with SR9009 or DMSO as vehicle control, before being subsequently exposed to IL-13 at hour 72. *N* = 3 per genotype per treatment group. (G – H) The average TEER for each experimental phase (hours 28–48, 52–72, 76–96) were calculated for each individual well. N = 3 per treatment group per experiment phase, analysed by two-way ANOVA with post-hoc Tukey's t-tests. Data shown as mean +/− SEM. (I) – Western Blots were performed to assess the relative quantity of ZO.1,Occludin, and NR1D1 proteins in lung tissue lysates taken from HDM exposed mice +/− SR9009 treatment. (J) - qPCR data showing the relative expression of tight junction gene *Zo.1* in lungs taken from HDM-exposed mice +/− treatment with SR9009. (K) - qPCR data showing the relative expression of tight junction gene *Ocln* in lungs taken from HDM-exposed mice +/− treatment with SR9009. (I—K) – *n* = 4 per treatment group. Data shown as mean +/− SEM, analysed by unpaired two-tailed t-tests. (L) Quantification of murine albumin concentrations in BALF samples from HDM and PBS treated wild type and CCSP-BMAL1 KO mice. *n* = 10 per genotype per treatment group, analysed by two-way ANOVA with post-hoc Tukey's t-tests. Data shown as mean +/− SEM.

Dosing ALI cultures with SR9009, a synthetic REVERBa ligand, increased TEER in both WT and CCSP-*Bmal1* KO groups, suggesting increased barrier integrity (Fig. 5E–H). Importantly, we note that whilst *Bmal1* ablation renders the circadian clock in the club cell non-functional, residual levels of REVERBa are still found in these cells,^26^ therefore we postulated that sufficient protein existed for the REVERBa ligand SR9009 to exert effect. Following SR9009 treatment, cells were exposed to IL-13, a cytokine associated with allergic inflammation and with barrier disruption in particular.44., 45 Whilst IL-13 exposure did reduce the TEER values in both WT and CCSP-*Bmal1* KO cultures, this was not by enough to reduce barrier integrity below baseline (Fig. 5E–H). Indeed, cultures exposed to IL-13, in the absence of SR9009 pre-treatment, exhibited a significant reduction in TEER below their original baseline values (Fig. 5E–H). Reversing the order of treatments further demonstrated that dosing epithelial cells with SR9009, post-IL-13 exposure, restored TEER values to baseline (Supp Fig. S5A–D).

Since HDM exposure is associated with reduced tight junction gene expression in-vivo (Supp Fig. S5E, F), we hypothesised based on our in-vitro experiments that treating HDM-exposed mice with SR9009 could help restore barrier integrity. Analysis of *Zo.1* and *Ocln* gene and protein expression revealed that SR9009 treatment was indeed able to increase the expression of these tight junction components in the lung tissue of mice exposed to HDM (Fig. 5I, J, K), concurrent with increasing NR1D1 protein expression levels (Fig. 5I).

Functionally, a reduction in the expression of airway epithelial tight junction components could precipitate increased airway leakiness. To assess this we analysed BALF samples for their albumin content (Fig. 5L). Since albumin is a serum protein, it is not expected to be found in the BALF at high concentrations and its accumulation in the BALF can be indicative of compromised barrier integrity.^46^, ^47^, ^48^ In particular, BALF albumin concentration is indicative of inside-out leakiness, which is the direction of leakiness that would most closely align with our hypotheses on immune cell trafficking being related to airway barrier integrity.^48^ In our analysis we found that whilst HDM-exposed mice exhibited increased BALF albumin concentrations, this increase was markedly greater in CCSP-BMAL1 KO mice (Fig. 5L**)**.

## Discussion

This study reveals that immune cell accumulation in the lungs and airways of mice with chronic AAD follows a 24-h rhythm. The club cell circadian clock plays a central role in coordinating this temporal variation. Additionally, we demonstrate that the club cell regulates rhythmicity in airway barrier permeability, likely via REVERBa, linking the circadian clock to barrier integrity.

A number of prior studies have demonstrated that the circadian clock plays a key role in regulating allergic airways disease. For example, it has been shown that global *Nr1d1*knockout mice exhibit exaggerated disease states following HDM-exposure compared to wild type mice^49^. Likewise, global *Bmal1* knockout mice have also been shown to exhibit aggravated inflammatory responses to pulmonary viral infections.^50^ Such studies, whilst they indeed draw associations between lung inflammation and the molecular circadian clock, lack a degree of mechanistic specificity implicating clocks in particular cell types as especially relevant.

In this study the club cell has emerged as a crucial cell type responsible for gating time-of-day responses to allergen challenge; similar responses to LPS and viral challenges have been reported previously.^25^, ^26^ We suggest that the club cell coordinates the time-of-day dependent changes in BALF eosinophils and TH-cell populations through regulating both the physical integrity of the airway barrier as well as the release of chemokines such as eotaxins which attract key leukocytes to the inflamed airway.51., 52 Indeed, we note that BALF eotaxin-1 concentrations peak in this model at ZT18, directly preceding the peak in BALF eosinophil accumulation at ZT0. Moreover, we confirm previously reported findings that circulating eosinophils peak in population size at ZT6,^9^, ^10^, ^11^, ^53^, ^54^ whilst we also found that eosinophil accumulation in peripheral lymphoid tissues, namely the spleen, peaked at the later ZT18 timepoint. Critically, in CCSP-BMAL1 KO mice, no time-of-day difference in either BALF eotaxin-1 or eotaxin-2 concentrations was detected. Whilst eotaxin-1 is largely produced by epithelial cells, eotaxin-2 is also produced by airway macrophages and this finding suggests a close relationship between these cell types to coordinate and synchronise time-of-day responses to allergic inflammation. This has been suggested before in models using myeloid BMAL1 deletion which found reduced eotaxin-1 production by airway epithelial cells.^55^ The seemingly bidirectional regulatory relationship between macrophages and the airway epithelium in the context of circadian disruption warrants further investigation.

Whilst we have observed that an epithelial circadian clock gates temporal variations in leukocyte accumulation in the lungs and BALF of HDM-exposed mice, other clocks in other cells must also be considered in the context of allergic airways disease. Notably, it has been shown that eosinophils and mast cells (two critical effector leukocytes driving asthma pathology in humans) possess functional molecular clocks of their own,^56^ in addition to exhibiting time-of-day dependent differences in their activation states.^56^ It is likely that circadian control of leukocyte trafficking and activity is therefore reliant on a complex circuit of cellular clocks acting in concert to drive pathology.

Additionally, we found that in wild type mice, serum IgE concentrations exhibited significant time-of-day dependent variation a phenomenon that failed to be recapitulated in CCSP-BMAL1 KO mice. We found that this variation in serum IgE was not correlated with increases in B-cell numbers in the peripheral blood, suggesting it was perhaps more so a result of heightened B-cell activity. It would be of interest therefore to pursue the role of circadian signalling in B-cell activation and immunoglobulin production in models of AAD.

We also confirm previously noted observations that the club cell specific *Bmal1* knockout mouse is associated with increased pulmonary neutrophilia, both at baseline and in cases of inflammatory lung disease.^25^ The mechanisms behind this may be related to the perturbed production of CXCL5 by airway epithelial cells, although this warrants further investigation.^25^, ^26^, ^57^, 58.

Clock-controlled regulation of tight junction genes (*Zo.1, Ocl*) has been reported in other tissues, and our findings extend this to the lung, suggesting that here the club cells of the airway epithelial barrier play a significant role in coordinating tight junction rhythmicity.20., 22. We, and others, have found that compounds that activate REVERBa such as SR9009, are able to reduce barrier permeability in ex-vivo experiments.59., 60. The ability of SR9009 to reduce barrier permeability, and thereby potentially limit the influx of allergens and activation of immune pathways perpetuating airway barrier damage, raises the potential of utilising inhaled clock-targeting drugs as novel asthma therapy. Currently, there are several agonists available that target components of the molecular circadian clock, many however have notable off-target effects. Whilst SR9009 has been used extensively in pre-clinical models to probe the impact of REVERB activation on processes underpinning inflammatory61., 62 and metabolic diseases,^63^, ^64^ it has been found to target metabolic pathways in liver cells even in the complete absence of REVERBa,^65^ indicating some off-target effects. Interestingly, SR10067 (another REVERBa agonist) as well as a CRY protein agonist, have both been shown to increase tight junction gene and protein expression in the lung, suggesting a mechanism involving the molecular clock.60., 66., 67.

These findings have significant implications for our understanding of allergic asthma. Firstly, we have previously shown that disease severity in HDM-exposed mice is linked to timing of allergen exposure,^32^ and results presented here suggest that this is likely due to changes in airway permeability over the 24 h day. Administering allergens when the barrier is more permeable may exacerbate inflammation, highlighting the need to consider dosing time in experiments. Secondly, increased permeability also facilitates leukocyte migration, potentially explaining time-of-day variations in leukocyte populations, particularly eosinophils, observed in BALF and patient sputum.^5^, 32. Finally, in line with findings here, time-of-day differences in CD4+ T-cell populations in allergen-challenged mice have been previously observed, but TH-cell subsets and the molecular clock's role remained unclear.^55^ We found that *Bmal1* ablation in club cells disrupted TH2 rhythmicity, while TH9 cells exhibited a phase shift and increased amplitude. This increase in TH9 cells in CCSP-B*mal1* KO mice was accompanied by elevated BALF IL-9 levels, especially at ZT12. IL-9 is a cytokine of particular importance in chronic allergic airways disease as it promotes mucus hypersecretion by goblet cells.^68^, 69. Indeed, we observed a concurrent increase in airway mucus at ZT12 in PAS-stained lung sections from CCSP-*Bmal1* KO mice compared to wildtype controls. TH9 cell differentiation relies on both IL-4 and TGF-β.^68^, 70. Whilst we did not observe any increase in IL-4 production in our current study, levels of TGF-β were not quantified and could potentially explain the noted increase in TH9 cells in CCSP-*Bmal1* KO mice. Further work to investigate the role of the club cell clock mechanism in regulating TH9/IL-9 pathways is warranted given the role of TH9 in asthma.^68^, 69., 71, 72

Additionally, we focussed specifically on how the club cell molecular clock regulates immune cell trafficking in AAD. CCSP-*Bmal1* KO mice exhibited loss of temporal variation in cellular composition of the BALF, with reduced inflammatory cell populations at ZT0 compared to WT littermates. Whilst in this study we identify a loss of time-of-day variation in CCSP-BMAL1 KO mice between ZT0 and ZT12, we cannot definitively rule out the possibility that temporal patterns were shifted to peak outside the sampling times used in this study. Future work should elucidate how this translates to alterations in lung function and thus the extent to which the club cell molecular clock controls daily rhythms in AHR.

The genetic knockout model used in the study, whilst targeting CCSP-expressing cells, the majority of which are club cells, may also have affecting BMAL1 expression in other epithelial cell types. It has been reported for example that the CCSP-iCRE utilised here specifically, also shows activity more widely across the mouse proximal airway epithelium.^73^ Furthermore, as club cells adapt to inflammatory conditions, they are capable of pathological transdifferentiation into goblet cells.74., 75 This would imply that the BMAL1 deficiency originating in a club cell, could potentially be passed on to goblet cells. Indeed, our histological findings revealed a loss of time-of-day dependent changes in airway mucus production, potentially linked to the emergence of BMAL1-deficient goblet cells.

In conclusion, we have shown that the club cell coordinates time-of-day dependent changes in eosinophil and TH-cell populations through regulating both the physical integrity of the airway barrier, and the release of chemokines. These findings have multiple clinical implications, emphasising the need to take time into consideration when diagnosing, assessing and treating allergic asthma. Furthermore, these data highlight the potential for clock-modulatory compounds as potential therapeutics in AAD.

## CRediT authorship contribution statement

**Jafar Cain:** Writing – review & editing, Writing – original draft, Project administration, Methodology, Investigation, Formal analysis, Data curation, Conceptualization. **Amlan Chakraborty:** Writing – review & editing, Writing – original draft, Methodology, Investigation, Formal analysis, Data curation, Conceptualization. **Vishvangi Deugi:** Writing – review & editing, Writing – original draft, Project administration, Investigation. **Karolina Krakowiak:** Writing – review & editing, Writing – original draft, Investigation. **David A. Bechtold:** Writing – review & editing, Writing – original draft, Supervision, Methodology, Formal analysis, Data curation, Conceptualization. **Julie E. Gibbs:** Writing – review & editing, Writing – original draft, Supervision, Resources, Project administration, Methodology, Investigation, Funding acquisition, Formal analysis, Data curation, Conceptualization. **Hannah J. Durrington:** Writing – review & editing, Writing – original draft, Visualization, Supervision, Resources, Project administration, Methodology, Investigation, Funding acquisition, Formal analysis, Data curation, Conceptualization.

## Funding

JC is funded by the Kennedy Trust for Rheumatology Research IMPACT MB-PhD Programme. HJD is funded by MRC-CSF-MR/V0229460/1 and Asthma and Lung UK WAPG22/100005. JEG is an Arthritis UK Senior Fellow (22625).

## Declaration of competing interest

The authors declare that they have no known competing financial interests or personal relationships that could have appeared to influence the work reported in this paper.
