# Supplementary material for: The club cell circadian clock regulates temporal patterns in leukocyte trafficking in chronic allergic airways disease
Source: Mucosal Immunol. 2026 Oct;19(5):None. doi: 10.1016/j.mucimm.2026.100384 (PMC13649646; doi:10.1016/j.mucimm.2026.100384)
Supplement: Supplementary file 1 [file mmc1.docx]

**Supplementary Figure 1**

(A-J) – Immune cell subsets from HDM/PBS treated mice were quantified by flow cytometry from BALF, lung and spleen samples taken on day 36 of the chronic HDM exposure protocol at 4 6-hourly intervals. (K – O) Quantification of cytokines (IL-2, IL-6, TNFa, KC, and MCP-1) in BALF samples taken on day 36 of the chronic HDM exposure protocol at 4 6-hourly intervals. (P) – Serum IgE was quantified by ELISA from peripheral blood samples taken from HDM/PBS treated mice on day 36 of the chronic HDM exposure protocol. (A-P) – All data presented as mean +/- SEM, analysed by one/two-way ANOVA as appropriate with post-hoc Sidak’s multiple comparison tests. N= 5 – 21.

**Supplementary Figure 2**

(A-I) – Immune cell subsets from HDM/PBS treated CCSP-BMAL1 KO mice and wild type littermate controls were quantified by flow cytometry from BALF, lung and spleen samples taken on day 36 of the chronic HDM exposure protocol at either ZT0 or ZT12. (A-I) – All data presented as mean +/- SEM, analysed by three-way ANOVA with post-hoc Sidak’s multiple comparison tests. N= 5 – 6. (J) – Serum IgE was quantified by ELISA from peripheral blood samples taken from HDM/PBS treated mice on day 36 of the chronic HDM exposure protocol from CCSP-BMAL1 KO mice and wild type littermate controls at either ZT0 or ZT12. Data presented as mean +/- SEM, analysed by three-way ANOVA with post-hoc Sidak’s multiple comparison tests, n= 4-5. (K) – Quantification of B-cells by flow cytometry in peripheral blood samples from HDM/PBS treated mice on day 36 of the chronic HDM exposure protocol from CCSP-BMAL1 KO mice and wild type littermate controls at either ZT0 or ZT12. Data presented as mean +/- SEM, analysed by three-way ANOVA with post-hoc Sidak’s multiple comparison tests, n= 5-6.

**Supplementary Figure 3**

(A-J) - Quantification of cytokines present in BALF samples of HDM/PBS treated CCSP-BMAL1 KO mice and wild type littermate controls taken at ZT0 and ZT12 on day 36 of the chronic HDM exposure protocol. All data presented as mean +/- SEM analysed by three-way ANOVA with post-hoc Šidak’s multiple comparison tests. N= 5-6. First level output of three-way ANOVA shown as Genotype / Time / Treatment.

**Supplementary Figure 4**

(A-D) – Representative immunohistochemical staining of airway epithelial cells in FFPE lung tissue samples stained for ZO.1 from PBS treated wild type mice at ZT0 (A), ZT6 (B), ZT12 (C), ZT18 (D). (E-H) Representative immunohistochemical staining of airway epithelial cells in FFPE lung tissue samples stained for Occludin from PBS treated wild type mice at ZT0 (E), ZT6 (F), ZT12 (G), ZT18 (H). (I) – Quantification of epithelial cells stained positive for ZO.1 across all 4 timepoints at the end of the chronic AAD protocol. N=6-12 per timepoint per treatment group, data shown as mean +/- SEM, analysed by two-way ANOVA with post-hoc Sidak’s multiple comparison tests. First level output of ANOVA shown as Interaction / Time / Treatment. (J) – Quantification of epithelial cells stained positive for Occludin across all 4 timepoints at the end of the chronic AAD protocol. N=6-12 per timepoint per treatment group, data shown as mean +/- SEM, analysed by two-way ANOVA with post-hoc Sidak’s multiple comparison tests. First level output of ANOVA shown as Interaction / Time / Treatment.

**Supplementary Figure 5**

(A - B) – Trans-epithelial Electrical Resistance measurements taken from wild type (A) and CCSP-BMAL1 KO (B) air-liquid interface airway epithelial cell cultures every 4 hours from 12 hours post-synchronisation of internal clocks using dexamethasone to 96 hours post-synchronisation. At hour 48 cultures were exposed to IL-13 prior to subsequently being dosed with SR9009 or DMSO as vehicle control at hour 72. N=3-6 per genotype per treatment group. (C – D) – Box and Whiskers plots showing the median and interquartile ranges of TEER recordings per each treatment group. N= 42 per treatment group, analysed by one-way ANOVA with post-hoc Dunnett’s multiple comparison tests. (E) - qPCR data showing the relative expression of tight junction gene *Zo.1* at ZT0, ZT6, ZT12, and ZT18 in whole lung samples taken from wildtype PBS/HDM-treated female mice. (F) - qPCR data showing the relative expression of tight junction gene *Occludin* at ZT0, ZT6, ZT12, and ZT18 in whole lung samples taken from wildtype PBS/HDM-treated female mice. (E-F) – qPCR data was normalised to the PBS-treated ZT0 group, and relative to the expression of housekeeping gene *Hprt,* shown as mean +/- SEM, analysed by two-way ANOVA with post-hoc Sidak’s multiple comparison tests, n= 5-6. First level output of two-way ANOVA shown as Interaction / Time / Treatment.

**Supplementary Figure 6**

(A) Immunophenotyping of T-cell subsets by flow cytometry: TH2, TH9 and T-reg cells were identified by flow cytometry. First, cells were selected based on forward and side scatter profiles. Singlet cells were then selected, following which a Live/Dead UV stain was used to exclude dead cells. TCRβ+ cells were then selected and taken forward to be gated CD4/CD8. TCRβ+ CD4+ cells were then analysed to show T-reg populations as TCRβ+ CD4+ CD25+ FOXP3+ cells. TCRβ+ CD4+ FOXP3- CD25- cells were then analysed to identify expression of transcription factors PU.1 (TH9 cells) and GATA3 (TH2) cells.

(B) The identification of granulocytes by flow cytometry: Eosinophil and neutrophil cells were detected by flow cytometry. First, cells were selected based on forward and side scatter profiles. Singlet cells were then selected, following which a Live/Dead UV stain was used to exclude dead cells. Subsequently cells were profiled in terms of LY6G expression, with LY6G+ cells being identified as neutrophils. The LY6G- population was further analysed to identify a LYG6- CD11c (mid) population and a LY6G- CD11c (low) population. LY6G- CD11c mid cells were further analysed in terms of SiglecF and CD11b expression. LY6G- CD11c mid SiglecF+ CD11b+ cells were then identified as inflammatory eosinophils (iEos). LY6G- CD11c low cells were analysed for their expression of SiglecF and CD11b with Ly6G- CD11c low SiglecF+ CD11b+ cells being identified as resident eosinophils (rEos).

**Supplementary Figure 7**

(A – D) Raw unprocessed images of immunoblots for the detection of ZO.1 (A), Alpha Tubulin (B), Occludin (C) and REVERBα (D).

**Supplementary Table 3**

| Antibody | Manufacturer | Clone | Concentration used for IHC |
| --- | --- | --- | --- |
| ZO.1 (Rabbit anti-mouse monoclonal) | Abcam | EPR19945-296 | 1:3000 |
| Occludin (Rabbit anti-mouse monoclonal) | Abcam | EPR20992 | 1:3000 |
| TSLP | EMD Millipore Corporation | ABT330 | 1:4000 |
